# Mechanisms limiting the long-term anabolic effects of teriparatide (PTH 1-34) on bone

**DOI:** 10.1093/jbmrpl/ziag093

**Published:** 2026-05-22

**Authors:** Clarissa Schmal, Joy Y Wu, Abbas Jafari

**Affiliations:** Division of Endocrinology, Department of Medicine, Stanford University School of Medicine, Stanford, CA 94305, United States; Department of Cellular and Molecular Medicine (ICMM), University of Copenhagen, DK-2200, Copenhagen, Denmark; Division of Endocrinology, Department of Medicine, Stanford University School of Medicine, Stanford, CA 94305, United States; Department of Cellular and Molecular Medicine (ICMM), University of Copenhagen, DK-2200, Copenhagen, Denmark

**Keywords:** teriparatide, PTH(1-34), osteoporosis, osteoprogenitor, anabolic window, Wnt, mechanical loading, PTH1R

## Abstract

Teriparatide, (recombinant human PTH 1-34) is an Food and Drug Administration (FDA)-approved anabolic therapy for osteoporosis. In patients with severe bone loss, daily teriparatide injections increase osteoblastic activity and bone turnover, leading to net gain in bone mass, increased BMD, and significantly reduced fracture risk. However, after 6-12 mo of treatment, its anabolic effects, reflected by increased serum bone turnover markers and LS-BMD gain, tend to diminish. Despite various efforts to address this challenge, the underlying mechanisms have remained poorly understood. This review discusses the main mechanisms proposed to limit teriparatide’s bone anabolic potential, including osteoprogenitor depletion, changes in bone remodeling dynamics, counter-regulatory molecules (eg, Wnt inhibitors), the influence of mechanical loading, and downstream signaling adaptations. Understanding these mechanisms is important for optimizing osteoporosis therapy and developing strategies to extend teriparatide’s “anabolic window.”

## Graphical Abstract

**Graphical Abstract:**
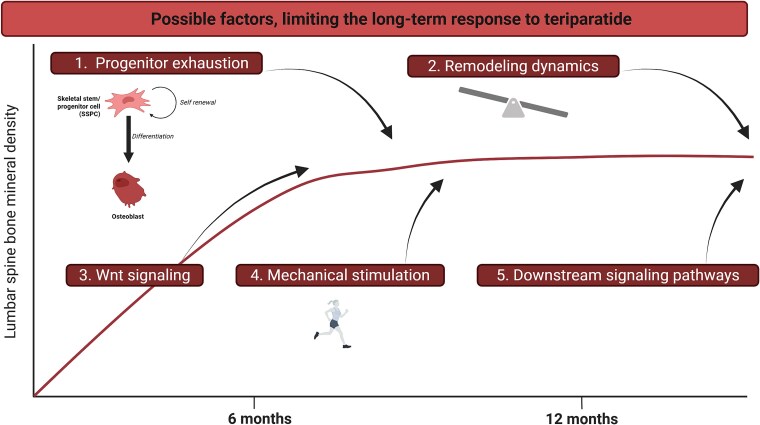

## The anabolic window: early gains and subsequent plateau

PTH, a peptide hormone produced by chief cells of the parathyroid gland, acts on bones, intestines and kidneys to regulate blood calcium levels, essential for muscle contraction, nerve impulse transmission, blood clotting, and skeletal mineralization.^1^ PTH stimulates bone remodeling (ie, increases both bone resorption and formation). However, it’s net effect on bone depends on the exposure kinetics: continuous PTH predominantly stimulates osteoclastic bone resorption leading to net bone loss, whereas pulsatile, intermittent PTH increases osteoblast number, bone formation, and net bone mass but also increases bone resorption.^2^^,^^3^ The period during which PTH-induced bone formation exceeds bone resorption is known as the “anabolic window.”

The first 34 N-terminal amino acids of PTH comprise the drug teriparatide. In 2002, teriparatide became the first osteoanabolic medication to receive approval from the Food and Drug Administration (FDA) for the treatment of osteoporosis. Teriparatide effectively reduces the risk of new vertebral fractures and increases BMD of the spine and hip in postmenopausal women and men with osteoporosis.^4^^,^^5^ Teriparatide is also approved for the treatment of glucocorticoid-induced osteoporosis.^6^

Current guidelines recommend teriparatide use for 2 yr.^7^^,^^8^ Although longer use of teriparatide can be considered if fracture risk remains high, the durability of its clinical benefits with extended therapy remains unclear, given the known waning anabolic response. The anabolic effects of teriparatide are most potent in the first 6-12 mo of administration. Afterwards, the rate of increase in BMD plateaus, as observed in multiple clinical trials.^4^^,^^9^^,^^10^ For example, in the Fracture Prevention Trial, 1637 postmenopausal women with osteoporosis were randomized to receive placebo or teriparatide (20 or 40 μg/d).^4^ In teriparatide-treated patients, LS-BMD increased rapidly for the first 6 mo, followed by slower gain from 6 to 18 mo.^11^

The European Study of Forsteo (EUROFORS) was an randomized controlled trial (RCT) designed to determine the effects of prior treatment on teriparatide efficacy and the effects of various sequential treatment regimens following 1-yr of teriparatide therapy. ΔLS-BMD in 865 patients receiving teriparatide (20 μg/d) was greatest for the initial 6 mo of treatment compared to the following 6-12 mo.^9^ In 503 patients who continued to receive teriparatide, the ΔLS-BMD continued to decline from 12 to 24 mo of treatment regardless of whether patients were previously treatment-naïve or treated with antiresorptives.^12^ In 245 patients treated with antiresorptive drugs prior to teriparatide, the same decline in ΔLS-BMD was demonstrated irrespective of the type of antiresorptive therapy.^13^

The TOWER trial investigated the effect of once weekly teriparatide (56.5 μg/wk) in 578 Japanese patients with osteoporosis. Here, LS-BMD increased most rapidly for the first 6 mo of treatment. Subsequently, ΔLS-BMD decreased from 6 to 12 mo followed by an additional decline from 12 to 18 mo.^10^ This characteristic pattern of early rapid BMD improvement followed by a plateau has been reproduced across diverse patient populations, pre-treatment regimens, teriparatide doses, and dosing intervals (Table 1).

**Table 1:** Clinical studies showing decreased ΔLS-BMD over time.

**Sex (F/M/both)**	**Study design, (study name)**	**Intervention**	**Study population (*n*** ^**a**^ **)**	**Time interval of maximum ΔLS-BMD**	**Reference**
F	RCT	40 μg/day s.c. TPTD + 200 μg nafarelin acetate	Women treated with nafarelin for endometriosis, *n* = 21	3-9 mo	^14^
	RCT (Fracture Prevention Trial)	20 μg/day s.c. TPTD	Postmenopausal, osteoporotic women, *n* = 541	0-6 mo	^4^ ^,^ ^11^
		40 μg/day s.c. TPTD	Postmenopausal, osteoporotic women, *n* = 552	0-6 mo	^4^ ^,^ ^11^
	RCT (EUROFORS)	20 μg/day TPTD	Postmenopausal, osteoporotic women, *n* = 865	0-6 mo	^9^
	RCT (DATA extension trial)	20 μg/day TPTD s.c.	Postmenopausal women with high fracture risk, *n* = 31	0-6 mo	^15^
		20 μg/day TPTD s.c. + 60 mg denosumab s.c.	Postmenopausal women with high fracture risk, *n* = 30	0-6 mo	^15^
	RCT	25 μg/day s.c. TPTD	Postmenopausal women, *n* = 43	0-12 mo	^16^
	Prospective observational	20 μg/day s.c. TPTD	Postmenopausal women, *n* = 109	0-12 mo	^17^
	RCT (PaVOS study)	20 μg/day s.c. TPTD + whole body vibration	Postmenopausal, osteoporotic women, *n* = 17	0-6 mo	^18^
		20 μg/day s.c. TPTD	Postmenopausal, osteoporotic women, *n* = 18	0-6 mo	^18^
	RCT (ACTIVE)	20 μg/day s.c. TPTD	Postmenopausal women, *n* = 818	0-6 mo	^19^
	RCT	20 μg/day s.c. TPTD	Postmenopausal women, *n* = 218	0-6 mo	^20^
	Prospective, observational	20 mg/day s.c. TPTD	Premenopausal women with idiopathic osteoporosis, *n* = 21	0-12 mo	^21^
	RCT	20 μg/day s.c. once-daily TPTD	Postmenopausal women, *n* = 51	0-6 mo	^22^
		28.2 μg/day s.c. twice weekly TPTD	Postmenopausal women, *n* = 51	0-6 mo	^22^
M	RCT	20 μg/day s.c. TPTD	Osteoporotic men, *n* = 151	0-3 mo	^5^
		40 μg/day s.c. TPTD	Osteoporotic men, *n* = 139	0-3 mo	^5^
	RCT	37 μg/day s.c. TPTD	Men with T-score of at least 2, *n* = 20	0-18 mo	^23^
	RCT	20 μg/day s.c. TPTD	Osteoporotic men, *n* = 9	0-12 mo	^24^
Both	RCT (TOWER trial)	56.5 μg/wk s.c. TPTD	Osteoporotic women, *n* = 273 Osteoporotic men, *n* = 13	0-6 mo	^10^
	RCT	20 μg/day s.c. TPTD	Patients with glucocorticoid-induced osteoporosis, *n* = 214	0-12 mo	^6^
	Prospective, observational	20 μg/day s.c. TPTD	Osteoporotic women, *n* = 52 Osteoporotic men, *n* = 8	0-12 mo	^25^

a Number of participants included in study arms treated with teriparatide.

Abbreviations: ΔLS-BMD, change in LS-BMD; RCT, randomized controlled trial; s.c., subcutaneous; TPTD, teriparatide.

Similar waning effects are observed in the impact of teriparatide on serum and urine bone turnover markers (Table 2). Within the first months of administration, teriparatide rapidly increases bone formation markers, such as bone-specific alkaline phosphatase (ALP), osteocalcin, type I collagen C-terminal propeptide, and type I collagen N-terminal propeptide (P1NP). Bone resorption markers such as crosslinked C-telopeptide of type I collagen, crosslinked N-telopeptide of type I collagen (NTX), and tartrate-resistance acid phosphatase type 5b (TRAP) increase with a delay.^34^^,^^35^ During long-term treatment with teriparatide, both bone formation and resorption markers peak after 6-12 mo followed by a decline.^36^

**Table 2:** Clinical studies showing progression of bone turnover markers over time.

**Markers**	**Intervention**	**No. of mo of treatment until peak concentration**	**Reference**
**Anabolic bone turnover markers**			
**Bone ALP, serum**	40 μg/day s.c. TPTD	6 mo	^14^
	40 μg/day s.c. TPTD	6 mo	^26^
	20 μg/day s.c. TPTD	12 mo	^5^
	40 μg/day s.c. TPTD	12 mo	^5^
	20 μg/day s.c. TPTD	12 mo	^27^
	37 μg/day s.c. TPTD	6 mo	^23^
	400 IU s.c. TPTD	9 mo	^28^
	40 μg/day s.c. TPTD	6 mo	^29^
**Osteocalcin, serum**	40 μg/day s.c. TPTD	9 mo	^14^
	20 μg/day s.c. TPTD	12 mo	^27^
	25 μg/day s.c. TPTD	9 mo	^16^
	30 μg/day s.c. TPTD	6 mo	^30^
	20 μg/day s.c. TPTD	6 mo	^15^
	S.c. TPTD	9 mo	^17^
	400 IU s.c. TPTD	12 mo	^28^
	25 μg/day s.c. TPTD	6 mo	^31^
**P1NP, serum**	20 μg/day s.c. TPTD	6 mo	^27^
	20 μg/day s.c. TPTD	6 mo	^32^
	25 μg/day s.c. TPTD	9-15 mo	^16^
	30 μg/day s.c. TPTD	6 mo	^30^
	20 μg/day s.c. TPTD	6 mo	^15^
	20 μg/day s.c. TPTD	6-12 mo	^33^
**PICP, serum**	20 μg/day s.c. TPTD	1 mo	^5^
	40 μg/day s.c. TPTD	1 mo	^5^
	400 IU, s.c. TPTD	6 mo	^28^
	20 μg/day s.c. TPTD	1 mo	^29^
	40 μg/day s.c. TPTD	1 mo	^29^
**Catabolic bone turnover markers**			
**NTX, urine**	40 μg/day s.c. TPTD	12 mo	^26^
	20 μg/day s.c. TPTD	12 mo	^5^
	40 μg/day s.c. TPTD	6 mo	^5^
	20 μg/day s.c. TPTD	6 mo	^32^
	20 μg/day s.c. TPTD	12 mo	^29^
	40 μg/day s.c. TPTD	12 mo	^29^
	25 μg/day s.c. TPTD	6 mo	^31^
**NTX, serum**	20 μg/day s.c. TPTD	6 mo	^27^
	30 μg/day s.c. TPTD	6 mo	^30^
**CTX, serum**	S.c. TPTD	6 mo	^17^
	20 μg/day s.c. TPTD	12 mo	^33^
	20 μg/day s.c. TPTD	12 mo	^15^
**DPD, urine**	40 μg/day s.c. TPTD	6 mo	^14^
	20 μg/day s.c. TPTD	6 mo	^5^
	40 μg/day s.c. TPTD	6 mo	^5^
	20 μg/day s.c. TPTD	12 mo	^29^
	40 μg/day s.c. TPTD	6 mo	^29^
**OHP, urine**	40 μg/day s.c. TPTD	6 mo	^14^
**PYD, urine**	400 IU s.c. TPTD	9 mo	^28^

Abbreviations: Bone ALP, bone alkaline phosphatase; CTX, cross-linked C-terminal telopeptide of type 1 collagen; DPD, deoxypyridinolone; NTX, cross-linked N-terminal telopeptide of type 1 collagen; OHP, hydroxyproline; PICP, serum procollagen I carboxy-terminal; PINP, serum procollagen I N-terminal; PYD, pyridinoline; s.c., subcutaneous; TPTD, teriparatide.

The waning effects of intermittent PTH treatment over time have also been reported in pre-clinical in vivo studies. In ovariectomized rats, PTH rapidly increased spine and whole skeleton BMD during the first 6 wk of treatment, after which the rate of BMD gain slowed. Serum markers of bone formation (ALP and osteocalcin) followed a similar trend.^37^ In another study, female ovariectomized or Sham-operated mice were treated with PTH for 8 wk. The percentage increase in trabecular volumetric BMD from pretreatment values was greater at 4 wk than 8 wk in both ovariectomized and Sham-operated mice.^38^ Another study evaluated the effect of PTH on ectopic ossicle formation in 4- to 6-wk-old nude mice after injection of BM stromal cells isolated from young C57BL/6 mice. Mice treated with PTH for 3 wk developed ossicles with increased radiopacity compared to controls, while mice treated for 7 wk had similar radiopacity irrespective of treatment.^39^ This demonstrates that increased bone formation at 3 wk was not sustained.

In this review, we will discuss different hypotheses for the waning effects of PTH(1-34) on bone, as evidenced by the reversal in serum bone turnover markers and stagnating ΔLS-BMD, including osteoprogenitor depletion, changes in bone remodeling dynamics, counter-regulatory molecules (Wnt inhibitors), the influence of mechanical stimulation, and downstream signaling adaptations.

## Gradual limitation of osteoprogenitor availability or responsiveness

Multiple tightly regulated mechanisms allow PTH(1-34) to increase the number of active osteoblasts.^40^ In skeletally mature bone, new osteoblasts are supplied by heterogeneous skeletal stem/progenitor cells (SSPCs) rather than by a single uniform progenitor pool that can be identified using a universal marker.^41^^,^^42^ It has been hypothesized that the waning anabolic effect of teriparatide may partly reflect a progressive limitation in the recruitment or osteogenic differentiation of these progenitor populations over time.

### Osteoprogenitor frequency limits the response to PTH(1-34)

Preclinical studies have shown that increased numbers of SSPCs and osteoprogenitors can enhance the anabolic effects of PTH(1-34).

Nuclear matrix protein 4 (Nmp4) also known as Cas-interacting zinc finger protein (CIZ) is a ubiquitously expressed transcription factor that shuttles between the cytoplasm and nucleus and has been implicated in restraining the anabolic response of bone to PTH.^43^ Global Nmp4 KO (Nmp4^(−/−)^) mice exhibit no major baseline skeletal abnormalities but show significantly greater trabecular bone gain in response to intermittent PTH(1-34) than WT mice.^44^^,^^45^ Notably, PTH-treated Nmp4^(−/−)^ mice had a 4-fold increase in CD45^−^/CD146^+^/CD105^+^/nestin^+^ BM stromal cells after 3 wk of PTH(1-34) treatment, and their BM produced 4 times more ALP^+^ colonies (CFU-Fs) in vitro than PTH-treated WT mice. These findings suggest that Nmp4/CIZ restricts the anabolic effects of PTH(1-34) by suppressing the size of the osteoprogenitor pool from which osteoblasts are recruited.^46^

Furthermore, conditional KO of Nmp4 in mesenchymal progenitors (using Prx1-Cre) but not in mature osteoblasts (Bglap-Cre) or osteocytes (Dmp1-Cre) enhanced the bone formation response to PTH(1-34), underscoring that the pool of early progenitors is essential for teriparatide’s full efficacy.^47^

Given that increased numbers of osteoprogenitors enhance the anabolic response to PTH(1-34) treatment, it is reasonable to hypothesize that gradual depletion of osteoprogenitors could contribute to limiting the anabolic response to PTH(1-34) over time.

### Effects of PTH(1-34) on osteoprogenitor proliferation and survival

Whether intermittent PTH(1-34) expands or depletes the osteoprogenitor pool depends on its influence on progenitor proliferation vs differentiation. If PTH primarily drives osteoprogenitors to differentiate into osteoblasts without replenishing them via proliferation, the progenitor pool could decline over time. Studies on this topic have yielded mixed results, as detailed in Table 3. The observed variability likely reflects differences in differentiation state, treatment pattern, and cell population. Among these, the studied osteoprogenitor population seems to be an especially important factor. Lineage tracing studies in mice have for example indicated increased proliferation of Gli+-metaphyseal mesenchymal progenitors^52^ but decreased proliferation of Sox9+-progenitors in response to short-term intermittent PTH(1-34).^59^ A limitation of these studies is that both Gli+-progenitors^63^ and Nes+-progenitors^50^ play a role in bone development and are known to decline with age. Besides, young, skeletally immature mice with a higher basal bone turnover rate were used, making it difficult to translate the results to an aged, human population. In this regard, although Sox9+ progenitors have been shown to contribute to osteoblast lineage cells in young adult mice, their specific role in aged, remodeling-dominant bone remains insufficiently defined. Additional studies are needed to define how prolonged PTH(1-34) administration affects osteoprogenitor populations relevant to bone remodeling in the mature and aging skeleton. Importantly, this should also be examined in patients receiving long-term teriparatide treatment to determine whether comparable changes occur in the clinical setting.

**Table 3:** The effects of intermittent PTH on osteoprogenitor proliferation and cell numbers.

**First author**	**Type of study**	**Intervention**	**Population**	**Outcome**	**Source**
**Increased osteoprogenitor proliferation with intermittent PTH**					
Di Benardo	Human, in vitro	Treating cultured cells with 10^−9^ M PTH for 3 d and 7 d.	Human mesenchymal stem cells from healthy donors	Increased proportion of cells in S-phase with no change in apoptosis rate	^48^
Ogita	Animal, in vivo	Single injection of PTH(1-34) (40 ng/g) and BrdU labeling	4-mo-old C57BL/6 mice.	Increase in number of BrdU labeled cells in PTH(1-34) treated mice	^49^
Méndez-Ferrer	Animal, in vivo	Intraperitoneal injection of PTH(1-34) (80 μg/kg/day) for 5 wk	Mice with cells expressing Nestin (Nes) tagged with green fluorescent protein (GFP), termed Nes-GFP mice. These cells are perivascular stromal cells, capable of forming CFU-Fs^50^	PTH(1-34)-treated Nes-GFP mice had a higher proportion of Nes-GFP+ cells in the S-phase of the cell cycle and a decreased proportion of these cells in G0/G1 phase compared to vehicle-treated Nes-GFP mice. PTH(1-34) doubled the number of bone marrow Nes-GFP cells.	^51^
Shi	Animal, in vivo	1. Tamoxifen activation followed by PTH(1-34) (400 ng/g) for 3 d and EdU labeling 2. Injecting PTH(1-34) for 3 d followed by tamoxifen activation	1-mo old Gli1-CreER^T2^;tdTomato mice, in which Gli1 positive metaphyseal mesenchymal progenitors (Gli + -MMPs) are marked with the red fluorescent protein tdTomato upon tamoxifen activation	1. PTH(1-34)-treated mice had an increased EdU+ tdTomato+/tdTomato+ ratio but constant rates of apoptosis compared to vehicle-treated mice 2. Increased numbers of Gli1+ cells, with increased EdU labeling indexes, indicating direct expansion of the Gli+-MMP population in response to PTH(1-34)	^52^
**Decreased or constant osteoprogenitor proliferation with intermittent PTH**					
Oniya	Animal, in vivo	Single injection of PTH(1-34) (80 μg/kg)	Young male Sprague-Dawley rats, isolation of distal femur 1 h post injection	Expression of early differentiation genes (c-fos, c-jun, c-myc) and IL-6 was increased, whereas histone H4 expression (a proliferation marker) was inhibited in distal femur bone	^53^
Dobnig	Animal, in vivo	1 wk of PTH(1-34) (8 μg/100 g) treatment and [3H]thymidine-labeling of dividing cells	16-mo-old female Sprague-Dawley rats	Only few osteoblasts originated from dividing cells or mitotic osteoprogenitors	^54^
Jilka	Animal, in vivo	Daily PTH(1-34) at 100 ng/g for 3 d and BrdU-labeling	3.6Col1A1 tk mice	PTH(1-34) treatment did not stimulate osteoprogenitor replication in cancellous or periosteal bone in mice	^55^
Wang	Animal, in vitro	Transient PTH(1-34) (25 nM) treatment for days 0-7 BrdU labeling Annexin V labeling	Calvarial cells isolated from mice	Decreased total DNA content at days 7 and 10 compared to control cells Inhibited cell proliferation (BrdU labeling) in pre-confluent cultures. No differences in confluent cultures Decrease in percentage of cells in S-phase on day 3 and reduced apoptosis (Annexin V)	^56^ ^57^
Balani	Animal, in vivo	1. Tamoxifen activation followed by daily PTH(1-34) (400 ng/g) for 7 d. EdU and Annexin V labeling 2. Daily PTH(1-34) for 7 or 21 d followed by tamoxifen activation	6-7 wk old Sox9-creERT2;R26RTomato mice. Sox9-expressing MSCs are found in the limb bud and differentiate into chondrocytes and osteoblasts^58^	1. Increase in Sox9+-cells in the metaphyseal spongiosa and cortical bone in PTH(1-34)-treated mice compared to control mice caused by reduced apoptosis (Annexin V) at constant rates of proliferation (EdU) 2. The metaphysis of PTH(1-34)-treated mice contained 141 Sox9+-cells after 7 d but only 75 Sox9+-cells after 21 d	^59^
Drake	Human, clinical	Daily teriparatide (40 μg/day) vs control treatment for 14 d	Hematopoietic lineage negative (lin^−^)/alkaline phosphatase positive (ALP^+^) osteoprogenitors isolated from the bone marrow of 20 (+19 control) postmenopausal women	No impact on proliferation markers, apoptosis markers, osteoblast differentiation markers and Wnt targets	^60^
**Progenitor cell numbers in response to PTH (combined effects on proliferation, differentiation and survival)**					
Nishida	Animal, in vivo	Daily PTH(1-34) (30 μg/kg) for 1 and 3 wk	Bone marrow cells from 9-wk-old, female Sprague-Dawley rats	Higher total numbers of CFU-F and higher numbers of CFU-F^ALP+^	^61^
Chen	Animal, in vivo	Intermittent PTH(1-34) (10 nM) for 6/48 h	Bone marrow stem cells isolated from long bones of 5-wk-old male Sprague-Dawley rats	Increased number of cells on days 3, 7, 10, and 14	^62^
Ogita	Animal in vitro	PTH(1-34) at high doses (5 × 10^−8^ M and 10^−7^ M)	Primary periosteal osteoblasts	Lower cell numbers on day 8 of treatment	^49^

### Effects of cyclic regimens, escalating regimens, and treatment holidays on the response to teriparatide

If the gradual depletion of osteoprogenitors contributes to the waning effects of teriparatide over time, cyclic regimens and treatment holidays might sustain the anabolic response. Cyclical teriparatide regimens and “drug holidays” have been explored as strategies to mitigate the plateau, although direct evidence of progenitor recovery is lacking. Several such regimens have been tested.

An initial clinical study in osteoporotic women on alendronate compared continuous daily teriparatide vs a cyclic regimen (3 mo on teriparatide, 3 mo off, repeated five times). In the continuously treated group, LS-BMD did not increase further from 12 to 15 mo, while the cyclically treated group had not yet reached a treatment plateau. With only 60% of the daily dose, the cyclic approach achieved 88.5% of the increase in BMD that the continuous regimen provided.^16^ Twenty-seven women, who continued to be at high risk of fracture after the 15 initial months of treatment, continued in a follow-up study, consisting of 12 mo of treatment with alendronate only, followed by 12 mo of retreatment with daily teriparatide and alendronate. Women experienced similar ΔLS-BMD in the retreatment period as in the first treatment course.^64^ Of note, concurrent treatment with alendronate has since been shown to limit the effectiveness of teriparatide,^30^ limiting the generalizability of the data to patients treated with teriparatide only. As this clinical study was underpowered for detecting changes in fracture risk, the group developed a mouse model. Mice were treated continuously for 7 wk or cyclically, alternating between PTH(1-34) injections and vehicle every week. Mice treated with the cyclic regimen achieved higher femur and vertebral BMD, osteocalcin concentration, and femur strength per unit PTH administered than mice treated with the continuous regimen.^65^ Microarchitectural parameters of the lumbar vertebrae improved equally in both groups, whereas the microarchitecture of the femur was affected to a larger extent by the continuous regimen.^66^ Overall, the mouse model indicated that cyclic PTH(1-34) regimens are reasonable alternatives to continuous regimens. Another study randomized postmenopausal women to constant (30 μg/day for 18 mo) vs escalating doses of teriparatide (20 μg/day for 6 mo, 30 μg/day for 6 mo, 40 μg/day for 6 mo). The escalating regimen prevented waning of bone formation markers during months 12-18, while achieving similar increases in BMD.^67^ It is possible that the escalating dose prevented a rapid depletion of osteoprogenitors.

On the contrary, other studies did not observe these positive effects of cyclic regimens. Women and men with osteoporosis were treated with teriparatide for 24 mo followed by 12 mo of treatment holiday and then treated with teriparatide again for another 12 mo. The response to teriparatide as measured by spine BMD and increase in osteocalcin, P1NP, and NTX remained attenuated in the second treatment period despite of the long treatment holiday.^68^ Similarly, another randomized trial compared the effects of daily vs cyclic (3 mo on, 3 mo off) teriparatide in treatment naïve women and women previously treated with alendronate. In treatment-naïve women, the daily regimen achieved a 2-fold greater gain in BMD than the cyclic group, while alendronate treated women had an equal increase in BMD in both regimens.^69^ Women with a T-score <2 after 2 yr of treatment with teriparatide, were invited to participate in a follow-up study. After 4 yr of cyclic treatment with teriparatide, a total of 24 mo on teriparatide over 8 cycles, there was no significant increase in BMD in women receiving a cyclic regimen compared to women receiving daily teriparatide for 2 yr followed by alendronate for 2 yr.^70^ While it could be argued that the chosen population consisted of low responders to teriparatide, the study did not find that cyclic treatment with teriparatide has advantages over continuous regimens.

Overall, some cyclic or dose-escalating teriparatide regimens can maintain bone formation and BMD gains longer, while other studies have not been able to replicate these benefits. Further research is needed to elucidate whether the success of some cyclic regimens is truly due to allowing osteoprogenitor recovery, or whether other factors (eg, re-sensitization of PTH signaling or re-balancing formation vs resorption) underlie the improved outcomes.

### Effects of aging on osteoprogenitor cells

Aging is associated with decreased numbers and proliferation potential of various osteoprogenitor populations.^71–73^ Moreover, aging reduces transcriptomic diversity of SSPCs and affects interactions of the skeletal and hematopoietic lineages in the bone marrow niche, increasing fragility.^74^ Therefore, older patients with a lower reserve of osteoprogenitors, may have a more limited response to teriparatide, especially if prolonged treatment further depletes these cells. A study treating aged mice with PTH(1-34) for 4 wk showed that aged mice experienced greater increase in spinal BMD and a larger increase in osteoblast number than young adult mice, indicating that age did not attenuate the anabolic response.^75^ In vitro PTH(1-34) treatment increased proliferation of MSCs isolated from younger human donors, while it had no effect on MSCs isolated from older donors, hinting that progenitors from elderly patients may be less responsive or fewer in number.^76^ Lastly, a meta-analysis of 15 clinical trials reported that the increase in spine BMD after teriparatide treatment was blunted by increasing age.^77^ However, this analysis could be confounded by age-related factors such as spinal osteoarthritis (which can artifactually affect BMD measurements). Overall, whether age-related osteoprogenitor decline limits teriparatide’s long-term efficacy remains uncertain. Additional studies are needed to clarify if older osteoporosis patients exhibit a faster waning of response due to diminished progenitor supply or altered PTH signaling.

### Future perspectives on the effects of PTH(1-34) on the osteoprogenitor cell population

In conclusion, there is currently no direct evidence that prolonged PTH(1-34) treatment truly depletes osteoprogenitors in vivo, nor that actively restoring the osteoprogenitor pool reverses the waning anabolic response. Future research into this important question must therefore address several key challenges. First, the pronounced heterogeneity within the SSPC landscape poses a challenge. Distinct human adult SSPC populations have been identified in specialized niches of long bones, including CD146+ cells in the bone marrow stroma and CD164+, CD73+, and PDPN+ cells in the periosteum and upper metaphysis.^78^^,^^79^ In addition, vertebral skeletal stem cells expressing XIC1 and PAX1 among others have been described.^80^ These populations of stem cells are further influenced by aging. CD200 has been proposed as a marker distinguishing fetal from adult stem cells.^74^ Further studies should therefore examine the effects of PTH(1-34) on these SSPC subsets in aged populations. Lineage tracing in studies in animal models can provide important mechanistic insides but must be long-term and focus on aged or osteoporotic animal models. Second, possible osteoprogenitor exhaustion through prolonged PTH(1-34) treatment might involve other aspects of osteoprogenitor physiology than proliferation such as altered fate regulation and senescence.^81^^,^^82^ The effect of long-term PTH(1-34) treatment on these factors should be considered in future studies.

## Bone remodeling dynamics and the coupling of resorption to formation

Another mechanism that may contribute to the waning anabolic effect of teriparatide is the altered coupling of bone formation and resorption during the remodeling process. Although teriparatide initially increases bone formation more than resorption, this imbalance is temporary. In clinical studies, bone formation markers rise early, whereas bone resorption markers increase later with continued treatment, narrowing the anabolic window.^34^

Evidence from combination therapy trials demonstrates that co-administration of denosumab (antibody targeting RANKL) with teriparatide can enhance or sustain BMD gains by mitigating resorption, underscoring the critical role of remodeling balance in determining the durability of the anabolic response.^83^ However, not all antiresorptives interact with PTH the same way, as for example, alendronate has been shown to limit teriparatide’s anabolic effect.^30^ In addition, the capacity to initiate new remodeling units may plateau over time,^84^ and osteoclast-driven feedback may further modulate the coupling process. During bone resorption, matrix-derived factors such as TGF-β are released^85^ and play a role in recruiting osteoblast progenitors to the bone surface, thereby coupling bone formation to resorption.^85^ However, TGF-β also inhibits osteoblast differentiation in vitro^86^^,^^87^ and in vivo^88^ and excessive TGF-β compromises skeletal homeostasis.^88^ In addition, elevations in serum calcium upon bone resorption may stimulate calcitonin secretion, which may indirectly restrain bone formation by suppressing osteoclast-derived pro-osteogenic coupling signals, such as sphingosine 1-phosphate.^89^ Together, these feedback mechanisms may limit further anabolic responses during prolonged teriparatide treatment.

Furthermore, prolonged PTH-induced remodeling can also increase cortical bone porosity, offsetting gains in trabecular compartments and limiting net improvements in BMD at certain sites.^90^^,^^91^

In summary, the balance of bone remodeling appears crucial: teriparatide’s early advantage (formation >> resorption) diminishes as resorption accelerates and catches up. Strategies that rebalance remodeling in favor of formation can prolong BMD gains, highlighting that the coupling between formation and resorption is a key determinant of the durability of teriparatide’s anabolic response.

## Wnt signaling antagonists

**Figure 1:**
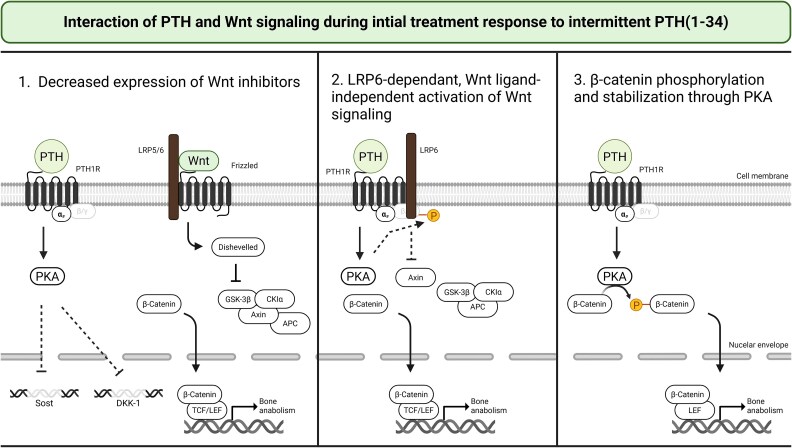
The interactions of PTH signaling and the Wnt signaling pathway. (1) PTH(1-34) interacts with its G-protein coupled receptor (PTH1R) and activates Gs leading to the activation of protein kinase A (PKA). PKA phosphorylates downstream targets (indicated by dashed lines) causing decreased expression of genes coding for the Wnt-antagonists sclerostin (SOST) and Dickkopf-protein 1 (DKK-1). In the absence of Wnt-antagonists, Wnt associates with frizzled receptors (Frizzled) and low-density lipoprotein receptor-related protein (LRP5/6). As a result, the β-catenin destruction complex (consisting of Axin, adenomatous polyposis coli (APC), and glycogen synthase kinase 3 β (GSK-3 β)) is disabled and β-catenin translocates to the nucleus and activates transcription of osteoanabolic genes. (2) PTH binds to PTH1R and activates PKA. PKA phosphorylates LRP6 and PTH-PTH1R forms a complex with phosphorylated LPR6, which recruits Axin and leads to inhibition of the β-catenin destruction complex and stabilization of β-catenin and its translocation to nucleus. (3) PKA phosphorylates β-catenin, leading to its stabilization and translocation to the nucleus. It should be noted that this figure only highlights the interactions between PTH and Wnt signaling pathways and does not depict the full range of mechanisms involved in bone anabolic effects of PTH. Figure is created using https://BioRender.com.

An important anabolic mechanism of teriparatide is the activation of the Wnt signaling pathway that promotes osteogenic differentiation. PTH affects the Wnt signaling pathway via at least 3 mechanisms.^92^ First, PTH has been shown to decrease the expression of Wnt signaling inhibitors. PTH receptor (PTH1R) is a G-protein coupled receptor that activates the G_s_ protein and protein kinase A (PKA).^93^ PKA phosphorylates downstream targets (eg, salt-induced kinase 2), causing histone deacetylases 4 and 5 to translocate to the nucleus and inhibit MEF2C-driven SOST expression, coding for the Wnt antagonist sclerostin.^94^ PTH can also decrease expression of the Wnt antagonist Dickkopf protein-1 (DKK-1).^95^ Second, PTH-PTH1R can also form a complex with low-density lipoprotein receptor-related protein 6 (LRP6) causing activation of canonical Wnt signaling.^96^ Lastly, it has been shown that PKA (upon activation by PTH/PTH1R) can phosphorylate and stabilize β-catenin.^95^ These 3 mechanisms (summarized in Figure 1) could be altered in long-term treatment with PTH. The following section focuses on the first mechanism as it has been suggested that a gradual increase in the expression of these Wnt antagonists during prolonged teriparatide treatment may limit activation of Wnt signaling and thereby contribute to the waning anabolic response to teriparatide.

### Wnt antagonists compensate for each other

Intriguingly, the Wnt antagonists DKK-1 and sclerostin have been shown to compensate for each other. DKK-1 inhibition increases expression of the Sost gene, coding for sclerostin, in mice. Moreover, DKK-1 antibody has potent bone anabolic effects in Sost^−/−^ mice, while only having negligible effects in Sost^+/+^ mice.^97^ Patients with deficient sclerostin production leading to sclerosteosis or Van Buchem disease, both have increased levels of DKK-1,^98^ and mice treated with sclerostin antibodies have increased DKK-1 protein levels.^99^^,^^100^ As PTH(1-34) affects the expression of these antagonists, a compensatory upregulation of Wnt antagonists over time could attenuate the anabolic effects of the drug.

### Effects of PTH(1-34) on DKK-1

Several clinical studies have measured DKK-1 levels during long-term teriparatide treatment, with somewhat variable results. Two prospective cohort studies observed that DKK-1 levels were increased as early as 6 mo of treatment and remained elevated after 18 mo compared to baseline.^101^^,^^102^ In contrast, another study showed that serum levels of DKK-1 first increased after 6 mo of treatment, at the same time as bone turnover markers started to stagnate.^103^ A third study found a slight decrease of DKK-1 after 6 mo, followed by a significant increase after 12 mo.^104^ Two of these studies also measured serum sclerostin levels and found these to be constant.^103^^,^^104^ Lastly, a study investigated the expression of serum microRNAs after 3 and 12 mo of treatment with teriparatide and discovered decreased expression of miR-33-3p, which negatively regulates DKK-1 expression.^105^

These observations raise the question of whether increased circulating DKK-1 contributes to attenuation of teriparatide efficacy over time. However, preclinical studies do not support this interpretation. Intermittent PTH has consistently been shown to suppress DKK-1 expression in vitro and in vivo, and in mice with osteoblast-targeted overexpression of DKK-1, intermittent PTH still produced a bone anabolic response comparable to that in wild-type mice.^106^ Thus, although clinical studies suggest that circulating DKK-1 may rise during long-term teriparatide treatment, current preclinical data indicate that this increase is unlikely to be a major driver of the waning anabolic response.

### Effects of PTH(1-34) on sclerostin

Several studies have monitored the serum levels of sclerostin in patients treated with teriparatide (Table 4), reporting variable results including increased, decreased and constant serum sclerostin levels in response to teriparatide treatment. In addition, a recent retrospective clinical study found that patients switched from teriparatide to romosozumab experienced further increases in LS, FN, and TH BMD, with greater gains than those switched in the reverse sequence.^113^ However, because the study did not include a comparator arm that continued teriparatide alone, it does not directly prove that sclerostin inhibition extends the anabolic window of teriparatide.

**Table 4:** Clinical studies investigating the effects of teriparatide on sclerostin serum levels.

**Study design**	**Intervention**	**Study population (*n*** ^**a**^ **)**	**Sclerostin serum concentrations**	**Prior osteoporosis medication**	**Blood sampling**	**Reference**
**Studies showing decrease of serum sclerostin**						
Prospective cohort study	20 μg/day s.c. TPTD for 18 mo	Postmenopausal osteoporotic women, *n* = 27	↓ at 6-9 mo and 12-18 mo	Oral bisphosphonates	At least 6 h after last s.c. injection	^107^
RCT	40 μg/day s.c. TPTD for 14 d	Postmenopausal women, *n* = 27	↓ at 14 d	Treatment naïve	Fasting morning samples	^108^
Prospective	56.5 μg/wk s.c. TPTD for 18 mo	Postmenopausal, osteoporotic women, *n* = 32	↓ at 12 and 18 mo	Treatment naïve	Fasting morning samples	^109^
RCT	20 μg/day s.c. TPTD for 18 mo	Postmenopausal, osteoporotic women, *n* = 5	↓ at 18 mo	Previously treated with alendronate	Fasting morning samples	^110^
**Studies showing constant serum sclerostin**						
RCT	20 μg/day s.c. TPTD for 18 mo	Postmenopausal women, *n* = 35	→ at 6, 12 and 18 mo	Unknown	Unknown	^103^
Retrospective, interventional	20 μg/day s.c. TPTD for 6 mo	Postmenopausal, osteoporotic women, *n* = 13	→ at 6 mo	Treatment naïve	Fasting morning samples	^111^
Prospective	20 μg/day s.c. TPTD for 12 mo	Postmenopausal, osteoporotic women, *n* = 20	→ at 3, 6, 9 and 12 mo	Treatment naïve	Unknown	^104^
RCT (PaVOS study)	20 μg/day TPTD + whole body vibration	Postmenopausal, osteoporotic women, *n* = 17	→ at 3 and 6 mo	5 women with previous bisphosphonate use	Fasting morning samples	^18^
	20 μg/day TPTD	Postmenopausal, osteoporotic women, *n* = 18	→ at 3 and 6 mo	10 women with previous bisphosphonate use	Fasting morning samples	^18^
**Studies showing increase in sclerostin**						
RCT (DATA trial)	20 μg/day s.c. TPTD for 24 mo	Postmenopausal, osteoporotic women, *n* = 27	↑ at 3, 6 mo, → at 12 mo compared to 6 mo	Treatment naïve within 6 mo of enrollment	Fasting morning samples	^112^
Prospective (MOAT study)	20 μg/day s.c. TPTD	Postmenopausal, osteoporotic women, *n* = 20	↑ at 12 mo	Bisphosphonate naïve	Fasting morning samples	^102^

a Number of participants included in study arms treated with teriparatide.

Abbreviations: ↓, decrease; →, constant level; ↑, increase; RCT, randomized controlled trial; s.c., subcutaneous; TPTD, teriparatide.

In preclinical studies, both increased and decreased levels of sclerostin can impact the anabolic effects of PTH(1-34) on bone. In transgenic mice, overexpressing Sost (SOST Tg) treated with intermittent PTH(1-34) for 2 mo, the increase in BMD in the LS, tibia, and distal femur was smaller in SOST Tg mice compared to WT mice. Moreover, the anabolic response to PTH(1-34) on trabecular bone volume fraction and trabecular thickness were attenuated in SOST Tg mice compared to WT mice. Dynamic bone histomorphometry revealed decreased bone formation rates in PTH(1-34)-treated SOST-Tg mice compared to PTH(1-34)-treated WT mice.^114^

PTH(1-34)-treated global Sost KO mice have an attenuated increase in BMD compared to PTH(1-34)-treated WT mice. While one study identified reduced bone formation rates as an underlying mechanism,^114^ another study found an increase in cortical porosity at constant cortical bone formation parameters.^115^ Importantly, PTH(1-34)-induced increase in trabecular bone volume fraction was not impaired in Sost KO mice compared to WT controls.^115^ Lastly, 2 studies on ovariectomized rats found that 12 wk of combined sclerostin antibody and PTH(1-34) treatment produced greater improvements in micro-CT-derived structural parameters, mechanical strength, and healing of a cylindrical bone defect than either monotherapy alone.^116^^,^^117^

Overall, the preclinical literature supports a biologically plausible role for sclerostin in modulating the anabolic response to intermittent PTH(1-34), and some clinical observations are compatible with this idea. However, it should be considered that the additive or synergistic skeletal effects of anti-sclerostin antibody combined with intermittent PTH(1-34) do not, by themselves, prove that sclerostin contributes to narrowing of teriparatide’s anabolic window. In addition, the human data are inconsistent, and definitive proof that rising sclerostin contributes to narrowing of the anabolic window during long-term teriparatide treatment is lacking. Notably, no clinical study has directly tested whether antagonizing sclerostin during ongoing teriparatide treatment can prevent or delay waning of the anabolic response.

### Future perspectives on the effect of Wnt signaling on teriparatide

Overall, current evidence does not support DKK-1 as a major driver of the waning anabolic effect of teriparatide, although circulating DKK-1 may increase during long-term treatment. By contrast, both preclinical and some clinical findings suggest that sclerostin may contribute to modulation of the anabolic response to PTH(1-34). In preclinical models, altered sclerostin levels affect the skeletal response to intermittent PTH, and combined PTH plus sclerostin antibody treatment produces greater anabolic effects than either monotherapy alone. Clinical evidence remains insufficient to establish causality, however, and no study has directly tested whether inhibiting sclerostin during continued teriparatide treatment can extend the anabolic window. Such a question would best be addressed in a head-to-head trial comparing teriparatide alone with teriparatide plus romosozumab after the initial treatment period. In addition, further mechanistic studies are needed because different skeletal cell populations may respond differently to PTH(1-34) and Wnt modulation, and compensatory interactions among Wnt pathway components may influence the overall outcome.

## Mechanical stimulation

The interaction between mechanical loading of the skeleton and PTH’s effects is another factor that may limit long-term bone anabolism. The mechanostat hypothesis, first described by Frost in 1987, proposed that bone mass is regulated by homeostatic feedback loops responding to mechanical strain. When the set-point for bone mass changes, there is an initial rapid adaptation of bone mass toward the new set-point, followed by stabilization at a higher level once the new steady-state is reached. In the case of teriparatide, treatment increases bone mass without a corresponding increase in mechanical usage (patients’ physical activity typically remains the same). As bone mass rises while mechanical load stays constant, mechanical strain per unit bone mass gradually decreases. This could trigger a feedback response that slows further bone gain—essentially, bone “senses” that it is now carrying the habitual loads more easily (lower strain) and thus reduces the stimulus for additional formation. It has been hypothesized that this mismatch between increased bone mass and unchanged mechanical strain may contribute to the drug’s waning effects.^118^ If so, one would predict that low mechanical strain (disuse or immobilization) would limit teriparatide’s anabolic action, whereas high mechanical strain (exercise or loading) would augment it.

In experimental settings, mechanical strain on bone comes from two sources: the strain generated by normal daily activities and additional externally applied loads (exercise regimens or device-based loading). At a given level of daily activity, a smaller bone mass experiences higher strain than a larger bone mass (since the same force is distributed over less area). Therefore, when teriparatide increases bone mass without a change in activity, the relative strain per bone decreases. If PTH’s efficacy is indeed strain-sensitive, then a scenario of low bone mass combined with external loading should yield the greatest response to PTH, while a high bone mass model with no mechanical loading (eg, immobilization) should yield the poorest response (Table 5). In support of this idea, it is shown that genetically high-bone-mass mice (sFRP1 or Sost KOs) show blunted responses to PTH, consistent with the notion that their bones, already strong and lightly strained under normal activity, get less benefit from PTH.^114^^,^^119^ The following section will investigate the effects of external loading on the effects of PTH(1-34).

**Table 5:** The expected responsiveness to PTH(1-34) from combined mechanical strain.

	**High bone mass**	**Low bone mass**
**External loading**	↑↑	↑↑↑↑↑
**Low external loading/immobilization**	↑	↑↑

### Effects of mechanical unloading vs loading on the anabolic response to PTH(1-34)

#### Unloading/immobilization

Several studies have examined if skeletal disuse attenuates the effects of PTH(1-34). One study subjected growing rats to 8 d of skeletal unloading with or without concurrent treatment with PTH(1-34). Rats experienced significant bone loss in response to unloading. While PTH(1-34) could not completely restore periosteal bone formation and overall tibial mass, skeletal unloading did not reduce the responsiveness of cancellous bone to PTH(1-34).^120^ Therefore, animals with low bone mass and low mechanical load still experienced a response to PTH(1-34). Another study immobilized mice by tail suspension for 8 or 15 d. Treatment with PTH(1-34) still had positive effects on bone volume fraction in the proximal tibia on day 8, while there were no effects on day 15. Moreover, PTH(1-34) treatment still increased expression of osteocalcin, osterix, and PTH1R expression on day 15.^121^ Lastly, a study investigated the effects of 2 wk of hindlimb unloading on adult male rats. PTH(1-34) treatment prevented bone loss in cancellous but not in cortical bone.^122^

PTH(1-34) treatment has also proven effective in treatment of immobilized patients. In patients with spinal cord injury, 12 mo of teriparatide therapy resulted in significant increases in spine aBMD compared with baseline.^123^ Another study compared the effects of teriparatide in patients with low levels of walking state to patients with high-level walking state. Here, the authors found similar increases in LS-BMD in both subgroups.^124^ Overall, these studies indicate that immobilization or unloading possibly reduces but does not abolish the osteoanabolic effects of PTH(1-34).

Consistent with the concept that mechanical input modulates the anabolic response to PTH, a remobilization study showed that in rats subjected to 19 wk of hindlimb immobilization, followed by continued immobilization or remobilization with concurrent PTH(1-34) treatment, remobilized limbs exhibited higher periosteal bone formation rates than immobilized limbs,^125^ indicating synergy between mechanical stimulation and PTH(1-34).

#### External loading

Several studies indicate that adding mechanical loading enhances PTH’s effects, particularly in cortical bone. For example, one study evaluated the effects of uniaxial compressive loading of the right tibia on PTH(1-34) treatment in ovariectomized mice. While there were no additive effects of the two treatments in cancellous bone, cortical thickness was higher with combination treatment than with PTH(1-34) treatment alone. The increased benefits were most prominent in those areas of the tibia subjected to higher strains under compressive loading.^126^

In 13-wk-old mice, combined mechanical stimulation and intermittent PTH(1-34) treatment for 6 wk yielded synergistic effects on tibial cortical bone volume.^127^ Moreover, 2 studies reported increased effects of combined compression of tail vertebrae and PTH(1-34) on bone formation rate in rats after a single injection,^128^ or after 2 and 4 wk of treatment.^129^ This result was confirmed in a recent study, showing additive effects of tail compression and PTH(1-34) on predicted strength and static morphometric parameters in the sixth caudal vertebrae of female mice.^130^ In rats subjected to overload of the right hindlimb and concurrent immobilization of the left hind limb, PTH(1-38) increased fracture load only in overloaded but not immobilized hindlimbs.^131^ In rats subjected to external loading by a 4-point bending device and PTH(1-34) for 3 wk, combined treatment resulted in synergistic effects on cortical bone formation surface, mineral apposition rate, and bone formation rate.^132^ These studies confirm the beneficial effects of high external loading in combination with PTH(1-34) on skeletal parameters.

Positive effects of mechanical stimulation have also been observed in clinical studies. One study treated 40 patients with teriparatide for 6 wk prior to hip replacement surgery and investigated the impact of the treatment on the 2 areas of the FN that undergo tension vs compression under normal daily activity. The endocortical bone formation rate was greater on the tensile region of the FN than on the compressed region of the FN, indicating that mechanical stimulation affects the anabolic response of bone to teriparatide.^133^ In addition, an RCT, comprising 35 postmenopausal women, reported increased effects of teriparatide on LS-BMD when combined with whole body vibration exercise for 12 mo.^18^

On the contrary, a study investigating the combined effect of mechanical loading and PTH(1-34) in aged 19-mo-old female mice found that PTH(1-34) abrogated the positive effects of loading on trabecular bone volume and trabecular thickness. In cortical bone, mechanical loading and PTH(1-34) had additive or independent effects on cortical thickness and porosity,^134^ suggesting that mechanical loading does not affect the bone anabolic response to PTH(1-34) in aged mice.

In conclusion, several studies have shown either additive or synergistic effects of concurrent external loading and PTH(1-34) treatment, while low mechanical strain seems to limit the effectiveness of PTH(1-34). These results support the hypothesis that lower mechanical strain could limit the long-term effects of PTH(1-34). However, aging may limit the sensitivity of bone to mechanical loading. Therefore, it remains unclear to what extent mechanical loading plays a role in the waning effects of teriparatide in older osteoporotic populations. From a practical standpoint, incorporating mechanical loading (eg, exercise) during teriparatide therapy may help to sustain its benefits, particularly in younger patients, although age-related declines in mechanosensitivity could dampen this advantage.

## Modification of downstream signaling pathways

The downstream signaling pathways of PTH are intricate and could be altered during long-term treatment with teriparatide.

PTH binds to PTH1R, a G-protein coupled receptor that activates G_s_ and G_q_ proteins. Once phosphorylated, PTH1R can recruit β-arrestin 2, leading to either dissociation from the G-protein and subsequent internalization of the β-arrestin-PTH1R complex or internalization of a β-arrestin-PTH1R-G-protein complex. The internalized complex can be either degraded or recycled.^93^ Additionally, the internalized β-arrestin-PTH1R-G-protein complex can continue to signal, prolonging the cAMP response.^135^ Several components of this downstream signaling pathway could contribute to the waning effects of teriparatide.

First, PTH1R could be affected by receptor downregulation or reduced receptor affinity in osteoprogenitors upon extended treatment with PTH. Pretreatment of primary calvarial cells with PTH(1-34) for 24-48 h downregulated PTH1R expression in vitro.^136^ Furthermore, case studies have reported acquired PTH resistance due to autoantibodies to PTH1R.^137^ However, this phenomenon has not been observed during teriparatide treatment.

Second, both insufficient and excessive desensitization could limit the bone-anabolic effects of teriparatide. Insufficient desensitization may prolong signaling, resulting in increased activation of bone resorption relative to formation, while excessive desensitization could reduce receptor availability. Repeat dosing of PTH(1-34) caused refractory cAMP responses to subsequent PTH(1-34) challenge in several studies.^138–140^ This desensitization involved internalization of the receptor,^139^ activation of kinases, such as PKA and PKC,^138^ and β-arrestin recruitment.^140^ The Na+/H+ exchange regulatory factor 1 (NHERF1) inhibited desensitization by preventing interaction with β-arrestin.^140^ Notably, PTH(1-34)-treated β-arrestin-2^(−/−)^ mice had higher periosteal bone formation rates compared to PTH(1-34)-treated β-arrestin-2^(+/+)^ mice, suggesting that limiting desensitization through β-arrestin inhibition may enhance the osteoanabolic effects of teriparatide.^141^ Conversely, abaloparatide, a PTH-related protein analogue, induced larger increases in cortical bone thickness compared to teriparatide in vivo, despite causing greater β-arrestin recruitment in vitro.^142^ This finding implies that β-arrestin recruitment may be positively associated with cortical bone gain. However, 2 other studies showed that abaloparatide causes less^143^ or similar^144^ β-arrestin recruitment compared to teriparatide in PTH1R-expressing HEK293 cells, indicating that the precise role of β-arrestin recruitment remains incompletely understood. In addition, the impact of long-term teriparatide treatment on β-arrestin recruitment has not been characterized further. Overall, the downstream signaling pathways activated by PTH warrant further investigation, at the preclinical and clinical level, in relation to the waning of its anabolic effects.

## Future perspectives and conclusion

This review aimed to explore different hypotheses proposed to explain the waning effects of teriparatide on bone. Several preclinical and clinical studies have attempted to overcome these waning effects, but the causes underlying the phenomenon remain partly understood. We discussed how osteoprogenitor supply, bone remodeling dynamics, counter-regulatory molecules (like Wnt inhibitors), mechanical feedback, and receptor signaling adaptations might contribute to the tapering of teriparatide’s efficacy. For each of these factors, we found only circumstantial or partial evidence of their role, rather than definitive proof. It is likely that the waning effect is multifactorial, with several of these mechanisms acting in concert.

Recently, an observational study suggested that patients previously treated with teriparatide have attenuated responses to the sclerostin antibody romosozumab.^145^ A preclinical study reported that osteoprogenitor frequency limited the effects of sclerostin antibodies,^146^ and another study showed dampened bone formation in response to repeat dosing with sclerostin antibody in mice concurrent with upregulation of Wnt antagonists.^147^ In addition, inhibition of DKK-1 showed a synergistic effect on bone formation induced by anti-sclerostin antibody.^100^ These parallel findings suggest that a shared adaptive mechanism (possibly depletion of a target cell population or induction of feedback inhibitors) may underlie the waning effectiveness of diverse anabolic agents. Identifying these common limiting factors is therefore of high significance for improving osteoporosis treatment.

In conclusion, continued research is needed to determine how to prevent or counteract the body’s adaptive responses to PTH, whether by combination therapies (eg, concurrent antiresorptives or Wnt signaling modulators), altered dosing schedules, or adjuvant mechanical interventions, to maintain bone formation at its peak. Unraveling the exact mechanisms behind the waning anabolic effect of PTH will enable the development of informed strategies to sustain bone gains and improve long-term outcomes for patients on osteoanabolic therapy.

## Data Availability

No new data were generated or analyzed in support of this research.

## References

[1] Peacock M . Calcium metabolism in health and disease. Clin J Am Soc Nephrol. 2010;5(Supplement_1):S23-S30. 10.2215/CJN.05910809 20089499

[2] Tam CS, Heersche JN, Murray TM, Parsons JA. Parathyroid hormone stimulates the bone apposition rate independently of its resorptive action: differential effects of intermittent and continuous administration. Endocrinology. 1982;110(2):506-512. 10.1210/endo-110-2-506 7056211

[3] Dobnig H, Turner RT. The effects of programmed administration of human parathyroid hormone fragment (1-34) on bone histomorphometry and serum chemistry in rats. Endocrinology. 1997;138(11, 11):4607-4612. 10.1210/endo.138.11.5505 9348185

[4] Neer RM, Arnaud CD, Zanchetta JR, et al. Effect of parathyroid hormone (1-34) on fractures and bone mineral density in postmenopausal women with osteoporosis. N Engl J Med. 2001;344(19):1434-1441. 10.1056/NEJM200105103441904 11346808

[5] Orwoll ES, Scheele WH, Paul S, et al. The effect of teriparatide [human parathyroid hormone (1-34)] therapy on bone density in men with osteoporosis. J Bone Miner Res. 2003;18(1):9-17. 10.1359/jbmr.2003.18.1.9 12510800

[6] Saag KG, Zanchetta JR, Devogelaer JP, et al. Effects of teriparatide versus alendronate for treating glucocorticoid-induced osteoporosis: thirty-six-month results of a randomized, double-blind, controlled trial. Arthritis Rheum. 2009;60(11):3346-3355. 10.1002/art.24879 19877063

[7] Forteo. Prescribing information. Eli Lilly and Company; 2026. Accessed June 3, 2026. https://pi.lilly.com/us/forteo-pi.pdf

[8] Dansk Endokrinologisk Selskab . Osteoporose National Behandlings Vejledning. Accessed June 3, 2026. https://endocrinology.dk/nbv/calcium-og-knoglemetabolisme/osteoporose/#elementor-toc__heading-anchor-11

[9] Minne H, Audran M, Simoes ME, et al. Bone density after teriparatide in patients with or without prior antiresorptive treatment: one-year results from the EUROFORS study. Curr Med Res Opin. 2008;24(11):3117-3128. 10.1185/03007990802466595 18838053

[10] Nakamura T, Sugimoto T, Nakano T, et al. Randomized Teriparatide [human parathyroid hormone (PTH) 1-34] Once-Weekly Efficacy Research (TOWER) trial for examining the reduction in new vertebral fractures in subjects with primary osteoporosis and high fracture risk. J Clin Endocrinol Metab. 2012;97(9):3097-3106. 10.1210/jc.2011-3479 22723322

[11] Marcus R, Wang O, Satterwhite J, Mitlak B. The skeletal response to teriparatide is largely independent of age, initial bone mineral density, and prevalent vertebral fractures in postmenopausal women with osteoporosis. J Bone Miner Res. 2003;18(1):18-23. 10.1359/jbmr.2003.18.1.18 12510801

[12] Obermayer-Pietsch BM, Marin F, McCloskey EV, et al. Effects of two years of daily teriparatide treatment on BMD in postmenopausal women with severe osteoporosis with and without prior antiresorptive treatment. J Bone Miner Res. 2008;23(10):1591-1600. 10.1359/jbmr.080506 18505369

[13] Boonen S, Marin F, Obermayer-Pietsch B, et al. Effects of previous antiresorptive therapy on the bone mineral density response to two years of teriparatide treatment in postmenopausal women with osteoporosis. J Clin Endocrinol Metab. 2008;93(3):852-860. 10.1210/jc.2007-0711 18160462

[14] Finkelstein JS, Klibanski A, Arnold AL, Toth TL, Hornstein MD, Neer RM. Prevention of estrogen deficiency-related bone loss with human parathyroid hormone-(1-34): a randomized controlled trial. JAMA. 1998;280(12):1067-1073. 10.1001/jama.280.12.1067 9757854

[15] Leder BZ, Tsai JN, Uihlein AV, et al. Two years of denosumab and teriparatide administration in postmenopausal women with osteoporosis (the DATA extension study): a randomized controlled trial. J Clin Endocrinol Metab. 2014;99(5):1694-1700. 10.1210/jc.2013-4440 24517156 PMC4010689

[16] Cosman F, Nieves J, Zion M, Woelfert L, Luckey M, Lindsay R. Daily and cyclic parathyroid hormone in women receiving alendronate. N Engl J Med. 2005;353(6):566-575. 10.1056/NEJMoa050157 16093465

[17] Anna G, Anne-Lise F, Clémence D, Jean-Michel P, Florence T. Factors associated with bone response to teriparatide in young postmenopausal women with osteoporosis. J Bone Miner Metab. 2023;41(2):278-285. 10.1007/s00774-023-01412-3 36894786

[18] Jepsen DB, Ryg J, Hansen S, Jørgensen NR, Gram J, Masud T. The combined effect of Parathyroid hormone (1-34) and whole-body Vibration exercise in the treatment of postmenopausal OSteoporosis (PaVOS study): a randomized controlled trial. Osteoporos Int. 2019;30(9):1827-1836. 10.1007/s00198-019-05029-z 31309239 PMC6717187

[19] Miller PD, Hattersley G, Riis BJ, et al. Effect of abaloparatide vs placebo on new vertebral fractures in postmenopausal women with osteoporosis: a randomized clinical trial. JAMA. 2016;316(7):722-733. 10.1001/jama.2016.11136 27533157

[20] Langdahl BL, Libanati C, Crittenden DB, et al. Romosozumab (sclerostin monoclonal antibody) versus teriparatide in postmenopausal women with osteoporosis transitioning from oral bisphosphonate therapy: a randomised, open-label, phase 3 trial. Lancet. 2017;390(10102):1585-1594. 10.1016/S0140-6736(17)31613-6 28755782

[21] Cohen A, Stein EM, Recker RR, et al. Teriparatide for idiopathic osteoporosis in premenopausal women: a pilot study. J Clin Endocrinol Metab. 2013;98(5):1971-1981. 10.1210/jc.2013-1172 23543660 PMC3644608

[22] Mochizuki T, Yano K, Ikari K, Okazaki K. Two-year outcomes of daily and twice-weekly teriparatide treatment in postmenopausal women with severe osteoporosis: a randomized non-blinded prospective study. J Bone Metab. 2024;31(2):162-168. 10.11005/jbm.2024.31.2.162 38886973 PMC11184152

[23] Finkelstein JS, Hayes A, Hunzelman JL, Wyland JJ, Lee H, Neer RM. The effects of parathyroid hormone, alendronate, or both in men with osteoporosis. N Engl J Med. 2003;349(13):1216-1226. 10.1056/NEJMoa035725 14500805

[24] Walker MD, Cusano NE, Sliney J Jr, et al. Combination therapy with risedronate and teriparatide in male osteoporosis. Endocrine. 2013;44(1):237-246. 10.1007/s12020-012-9819-4 23099796

[25] Stroup JS, Rivers SM, Abu-Baker AM, Kane MP. Two-year changes in bone mineral density and T scores in patients treated at a pharmacist-run teriparatide clinic. Pharmacotherapy. 2007;27(6):779-788. 10.1592/phco.27.6.779 17542760

[26] Body JJ, Gaich GA, Scheele WH, et al. A randomized double-blind trial to compare the efficacy of teriparatide [recombinant human parathyroid hormone (1-34)] with alendronate in postmenopausal women with osteoporosis. J Clin Endocrinol Metab. 2002;87(10):4528-4535. 10.1210/jc.2002-020334 12364430

[27] Ettinger B, San Martin J, Crans G, Pavo I. Differential effects of teriparatide on BMD after treatment with raloxifene or alendronate. J Bone Miner Res. 2004;19(5):745-751. 10.1359/JBMR.040117 15068497

[28] Kurland ES, Cosman F, McMahon DJ, Rosen CJ, Lindsay R, Bilezikian JP. Parathyroid hormone as a therapy for idiopathic osteoporosis in men: effects on bone mineral density and bone markers. J Clin Endocrinol Metab. 2000;85(9):3069-3076. 10.1210/jcem.85.9.6818 10999788

[29] Chen P, Satterwhite JH, Licata AA, et al. Early changes in biochemical markers of bone formation predict BMD response to teriparatide in postmenopausal women with osteoporosis. J Bone Miner Res. 2005;20(6):962-970. 10.1359/JBMR.050105 15883636

[30] Finkelstein JS, Wyland JJ, Lee H, Neer RM. Effects of teriparatide, alendronate, or both in women with postmenopausal osteoporosis. J Clin Endocrinol Metab. 2010;95(4):1838-1845. 10.1210/jc.2009-1703 20164296 PMC2853981

[31] Lindsay R, Nieves J, Formica C, et al. Randomised controlled study of effect of parathyroid hormone on vertebral-bone mass and fracture incidence among postmenopausal women on oestrogen with osteoporosis. Lancet. 1997;350(9077):550-555. 10.1016/S0140-6736(97)02342-8 9284777

[32] Arlot M, Meunier PJ, Boivin G, et al. Differential effects of teriparatide and alendronate on bone remodeling in postmenopausal women assessed by histomorphometric parameters. J Bone Miner Res. 2005;20(7):1244-1253. 10.1359/JBMR.050309 15940379

[33] Stepan JJ, Burr DB, Li J, et al. Histomorphometric changes by teriparatide in alendronate-pretreated women with osteoporosis. Osteoporos Int. 2010;21(12):2027-2036. 10.1007/s00198-009-1168-7 20135094

[34] Glover SJ, Eastell R, McCloskey EV, et al. Rapid and robust response of biochemical markers of bone formation to teriparatide therapy. Bone. 2009;45(6):1053-1058. 10.1016/j.bone.2009.07.091 19679211

[35] Pazianas M . Anabolic effects of PTH and the 'anabolic window'. Trends Endocrinol Metab. 2015;26(3):111-113. 10.1016/j.tem.2015.01.004 25662368

[36] Tabacco G, Bilezikian JP. Osteoanabolic and dual action drugs. Br J Clin Pharmacol. 2019;85(6):1084-1094. 10.1111/bcp.13766 30218587 PMC6533433

[37] Mitlak BH, Burdette-Miller P, Schoenfeld D, Neer RM. Sequential effects of chronic human PTH (1-84) treatment of estrogen-deficiency osteopenia in the rat. J Bone Miner Res. 1996;11(4):430-439. 10.1002/jbmr.5650110403 8992873

[38] Andersson N, Lindberg MK, Ohlsson C, Andersson K, Ryberg B. Repeated in vivo determinations of bone mineral density during parathyroid hormone treatment in ovariectomized mice. J Endocrinol. 2001;170(3):529-537. 10.1677/joe.0.1700529 11524233

[39] Pettway GJ, Schneider A, Koh AJ, et al. Anabolic actions of PTH (1-34): use of a novel tissue engineering model to investigate temporal effects on bone. Bone. 2005;36(6):959-970. 10.1016/j.bone.2005.02.015 15878317

[40] Jilka RL . Molecular and cellular mechanisms of the anabolic effect of intermittent PTH. Bone. 2007;40(6):1434-1446. 10.1016/j.bone.2007.03.017 17517365 PMC1995599

[41] Jeffery EC, Mann TLA, Pool JA, Zhao Z, Morrison SJ. Bone marrow and periosteal skeletal stem/progenitor cells make distinct contributions to bone maintenance and repair. Cell Stem Cell. 2022;29(11):1547-1561 e6. 10.1016/j.stem.2022.10.002 36272401

[42] Ambrosi TH, Sinha R, Steininger HM, et al. Distinct skeletal stem cell types orchestrate long bone skeletogenesis. Elife. 2021;10:e66063. 10.7554/eLife.66063 PMC8289409 34280086

[43] Bidwell JP, Childress P, Alvarez MB, et al. Nmp4/CIZ closes the parathyroid hormone anabolic window. Crit Rev Eukaryot Gene Expr. 2012;22(3):205-218. 10.1615/critreveukargeneexpr.v22.i3.40 23140162 PMC3586259

[44] Robling AG, Childress P, Yu J, et al. Nmp4/CIZ suppresses parathyroid hormone-induced increases in trabecular bone. J Cell Physiol. 2009;219(3):734-743. 10.1002/jcp.21717 19189321 PMC2746029

[45] Childress P, Philip BK, Robling AG, et al. Nmp4/CIZ suppresses the response of bone to anabolic parathyroid hormone by regulating both osteoblasts and osteoclasts. Calcif Tissue Int. 2011;89(1):74-89. 10.1007/s00223-011-9496-y 21607813 PMC3200195

[46] He Y, Childress P, Hood M Jr, et al. Nmp4/CIZ suppresses the parathyroid hormone anabolic window by restricting mesenchymal stem cell and osteoprogenitor frequency. Stem Cells Dev. 2013;22(3):492-500. 10.1089/scd.2012.0308 22873745 PMC3549622

[47] Atkinson EG, Adaway M, Horan DJ, et al. Conditional loss of Nmp4 in mesenchymal stem progenitor cells enhances PTH-induced bone formation. J Bone Miner Res. 2023;38(1):70-85. 10.1002/jbmr.4732 36321253 PMC9825665

[48] Di Bernardo G, Galderisi U, Fiorito C, et al. Dual role of parathyroid hormone in endothelial progenitor cells and marrow stromal mesenchymal stem cells. J Cell Physiol. 2010;222(2):474-480. 10.1002/jcp.21976 19918796

[49] Ogita M, Rached MT, Dworakowski E, Bilezikian JP, Kousteni S. Differentiation and proliferation of periosteal osteoblast progenitors are differentially regulated by estrogens and intermittent parathyroid hormone administration. Endocrinology. 2008;149(11):5713-5723. 10.1210/en.2008-0369 18617606 PMC2584601

[50] Ono N, Ono W, Mizoguchi T, Nagasawa T, Frenette PS, Kronenberg HM. Vasculature-associated cells expressing nestin in developing bones encompass early cells in the osteoblast and endothelial lineage. Dev Cell. 2014;29(3):330-339. 10.1016/j.devcel.2014.03.014 24823376 PMC4083679

[51] Mendez-Ferrer S, Michurina TV, Ferraro F, et al. Mesenchymal and haematopoietic stem cells form a unique bone marrow niche. Nature. 2010;466(7308):829-834. 10.1038/nature09262 20703299 PMC3146551

[52] Shi Y, Liao X, Long JY, et al. Gli1(+) progenitors mediate bone anabolic function of teriparatide via Hh and Igf signaling. Cell Rep. 2021;36(7):109542. 10.1016/j.celrep.2021.109542 34407400 PMC8432334

[53] Onyia JE, Bidwell J, Herring J, Hulman J, Hock JM. In vivo, human parathyroid hormone fragment (hPTH 1-34) transiently stimulates immediate early response gene expression, but not proliferation, in trabecular bone cells of young rats. Bone. 1995;17(5):479-484. 10.1016/8756-3282(95)00332-2 8579960

[54] Dobnig H, Turner RT. Evidence that intermittent treatment with parathyroid hormone increases bone formation in adult rats by activation of bone lining cells. Endocrinology. 1995;136(8):3632-3638. 10.1210/endo.136.8.7628403 7628403

[55] Jilka RL, O'Brien CA, Ali AA, Roberson PK, Weinstein RS, Manolagas SC. Intermittent PTH stimulates periosteal bone formation by actions on post-mitotic preosteoblasts. Bone. 2009;44(2):275-286. 10.1016/j.bone.2008.10.037 19010455 PMC2655212

[56] Wang YH, Liu Y, Buhl K, Rowe DW. Comparison of the action of transient and continuous PTH on primary osteoblast cultures expressing differentiation stage-specific GFP. J Bone Miner Res. 2005;20(1):5-14. 10.1359/JBMR.041016 15619664

[57] Wang YH, Liu Y, Rowe DW. Effects of transient PTH on early proliferation, apoptosis, and subsequent differentiation of osteoblast in primary osteoblast cultures. Am J Physiol Endocrinol Metab. 2007;292(2):E594-E603. 10.1152/ajpendo.00216.2006 17032929

[58] Akiyama H, Kim JE, Nakashima K, et al. Osteo-chondroprogenitor cells are derived from Sox9 expressing precursors. Proc Natl Acad Sci USA. 2005;102(41):14665-14670. 10.1073/pnas.0504750102 16203988 PMC1239942

[59] Balani DH, Ono N, Kronenberg HM. Parathyroid hormone regulates fates of murine osteoblast precursors in vivo. J Clin Invest. 2017;127(9):3327-3338. 10.1172/JCI91699 28758904 PMC5669555

[60] Drake MT, Srinivasan B, Mödder UI, et al. Effects of intermittent parathyroid hormone treatment on osteoprogenitor cells in postmenopausal women. Bone. 2011;49(3):349-355. 10.1016/j.bone.2011.05.003 21600325 PMC3143310

[61] Nishida S, Yamaguchi A, Tanizawa T, et al. Increased bone formation by intermittent parathyroid hormone administration is due to the stimulation of proliferation and differentiation of osteoprogenitor cells in bone marrow. Bone. 1994;15(6):717-723. 10.1016/8756-3282(94)90322-0 7873302

[62] Chen B, Lin T, Yang X, Li Y, Xie D, Cui H. Intermittent parathyroid hormone (1-34) application regulates cAMP-response element binding protein activity to promote the proliferation and osteogenic differentiation of bone mesenchymal stromal cells, via the cAMP/PKA signaling pathway. Exp Ther Med. 2016;11(6):2399-2406. 10.3892/etm.2016.3177 27284327 PMC4887846

[63] Shi Y, He G, Lee WC, McKenzie JA, Silva MJ, Long F. Gli1 identifies osteogenic progenitors for bone formation and fracture repair. Nat Commun. 2017;8(1):2043. 10.1038/s41467-017-02171-2 29230039 PMC5725597

[64] Cosman F, Nieves JW, Zion M, Barbuto N, Lindsay R. Retreatment with teriparatide one year after the first teriparatide course in patients on continued long-term alendronate. J Bone Miner Res. 2009;24(6):1110-1115. 10.1359/jbmr.081250 19113926 PMC2683649

[65] Iida-Klein A, Hughes C, Lu SS, et al. Effects of cyclic versus daily hPTH(1-34) regimens on bone strength in association with BMD, biochemical markers, and bone structure in mice. J Bone Miner Res. 2006;21(2):274-282. 10.1359/JBMR.051017 16418783

[66] Iida-Klein A, Lu SS, Cosman F, Lindsay R, Dempster DW. Effects of cyclic vs. daily treatment with human parathyroid hormone (1-34) on murine bone structure and cellular activity. Bone. 2007;40(2):391-398. 10.1016/j.bone.2006.09.010 17056311

[67] Yu EW, Neer RM, Lee H, et al. Time-dependent changes in skeletal response to teriparatide: escalating vs. constant dose teriparatide (PTH 1-34) in osteoporotic women. Bone. 2011;48(4):713-719. 10.1016/j.bone.2010.11.012 21111078 PMC3073572

[68] Finkelstein JS, Wyland JJ, Leder BZ, et al. Effects of teriparatide retreatment in osteoporotic men and women. J Clin Endocrinol Metab. 2009;94(7):2495-2501. 10.1210/jc.2009-0154 19401368 PMC2708954

[69] Cosman F, Nieves JW, Zion M, et al. Daily or cyclical teriparatide treatment in women with osteoporosis on no prior therapy and women on alendronate. J Clin Endocrinol Metab. 2015;100(7):2769-2776. 10.1210/jc.2015-1715 25961136 PMC5393523

[70] Cosman F, Nieves JW, Roimisher C, et al. Administration of teriparatide for four years cyclically compared to two years daily in treatment naive and alendronate treated women. Bone. 2019;120:246-253. 10.1016/j.bone.2018.10.020 30355512

[71] Nishida S, Endo N, Yamagiwa H, Tanizawa T, Takahashi HE. Number of osteoprogenitor cells in human bone marrow markedly decreases after skeletal maturation. J Bone Miner Metab. 1999;17(3):171-177. 10.1007/s007740050081 10757676

[72] Stenderup K, Justesen J, Clausen C, Kassem M. Aging is associated with decreased maximal life span and accelerated senescence of bone marrow stromal cells. Bone. 2003;33(6):919-926. 10.1016/j.bone.2003.07.005 14678851

[73] Kim HN, Chang J, Shao L, et al. DNA damage and senescence in osteoprogenitors expressing Osx1 may cause their decrease with age. Aging Cell. 2017;16(4):693-703. 10.1111/acel.12597 28401730 PMC5506444

[74] Ambrosi TH, Marecic O, McArdle A, et al. Aged skeletal stem cells generate an inflammatory degenerative niche. Nature. 2021;597(7875):256-262. 10.1038/s41586-021-03795-7 34381212 PMC8721524

[75] Knopp E, Troiano N, Bouxsein M, et al. The effect of aging on the skeletal response to intermittent treatment with parathyroid hormone. Endocrinology. 2005;146(4):1983-1990. 10.1210/en.2004-0770 15618351

[76] Zhou S, Bueno EM, Kim SW, et al. Effects of age on parathyroid hormone signaling in human marrow stromal cells. Aging Cell. 2011;10(5):780-788. 10.1111/j.1474-9726.2011.00717.x 21518242 PMC3158270

[77] Schwarz P, Jorgensen NR, Mosekilde L, Vestergaard P. Effects of increasing age, dosage, and duration of PTH treatment on BMD increase--a meta-analysis. Calcif Tissue Int. 2012;90(3):165-173. 10.1007/s00223-011-9564-3 22237954

[78] Chan CKF, Gulati GS, Sinha R, et al. Identification of the human skeletal stem cell. Cell. 2018;175(1):43-56.e21. 10.1016/j.cell.2018.07.029 30241615 PMC6400492

[79] Melis S, Trompet D, Chagin AS, Maes C. Skeletal stem and progenitor cells in bone physiology, ageing and disease. Nat Rev Endocrinol. 2025;21(3):135-153. 10.1038/s41574-024-01039-y 39379711

[80] Sun J, Hu L, Bok S, et al. A vertebral skeletal stem cell lineage driving metastasis. Nature. 2023;621(7979):602-609. 10.1038/s41586-023-06519-1 37704733 PMC10829697

[81] Wolfel EM, Fernandez-Guerra P, Norgard MO, et al. Senescence of skeletal stem cells and their contribution to age-related bone loss. Mech Ageing Dev. 2024;221(Special issue on aging and osteoporosis):111976. 10.1016/j.mad.2024.111976 39111640

[82] Fan Y, Hanai JI, Le PT, et al. Parathyroid hormone directs bone marrow mesenchymal cell fate. Cell Metab. 2017;25(3):661-672. 10.1016/j.cmet.2017.01.001 28162969 PMC5342925

[83] Tsai JN, Uihlein AV, Lee H, et al. Teriparatide and denosumab, alone or combined, in women with postmenopausal osteoporosis: the DATA study randomised trial. Lancet. 2013;382(9886):50-56. 10.1016/S0140-6736(13)60856-9 23683600 PMC4083737

[84] Dempster DW, Zhou H, Recker RR, et al. A longitudinal study of skeletal histomorphometry at 6 and 24 months across four bone envelopes in postmenopausal women with osteoporosis receiving teriparatide or zoledronic acid in the SHOTZ trial. J Bone Miner Res. 2016;31(7):1429-1439. 10.1002/jbmr.2804 26841258

[85] Tang Y, Wu X, Lei W, et al. TGF-beta1-induced migration of bone mesenchymal stem cells couples bone resorption with formation. Nat Med. 2009;15(7):757-765. 10.1038/nm.1979 19584867 PMC2727637

[86] Alliston T, Choy L, Ducy P, Karsenty G, Derynck R. TGF-beta-induced repression of CBFA1 by Smad3 decreases cbfa1 and osteocalcin expression and inhibits osteoblast differentiation. EMBO J. 2001;20(9):2254-2272. 10.1093/emboj/20.9.2254 11331591 PMC125448

[87] Maeda S, Hayashi M, Komiya S, Imamura T, Miyazono K. Endogenous TGF-beta signaling suppresses maturation of osteoblastic mesenchymal cells. EMBO J. 2004;23(3):552-563. 10.1038/sj.emboj.7600067 14749725 PMC1271802

[88] Grafe I, Yang T, Alexander S, et al. Excessive transforming growth factor-beta signaling is a common mechanism in osteogenesis imperfecta. Nat Med. 2014;20(6):670-675. 10.1038/nm.3544 24793237 PMC4048326

[89] Keller J, Catala-Lehnen P, Huebner AK, et al. Calcitonin controls bone formation by inhibiting the release of sphingosine 1-phosphate from osteoclasts. Nat Commun. 2014;5:5215. 10.1038/ncomms6215 25333900 PMC4205484

[90] Tsai JN, Uihlein AV, Burnett-Bowie SA, et al. Comparative effects of teriparatide, denosumab, and combination therapy on peripheral compartmental bone density, microarchitecture, and estimated strength: the DATA-HRpQCT study. J Bone Miner Res. 2015;30(1):39-45. 10.1002/jbmr.2315 25043459 PMC4396184

[91] Zebaze R, Takao-Kawabata R, Peng Y, et al. Increased cortical porosity is associated with daily, not weekly, administration of equivalent doses of teriparatide. Bone. 2017;99:80-84. 10.1016/j.bone.2017.03.042 28323145

[92] Baron R, Kneissel M. WNT signaling in bone homeostasis and disease: from human mutations to treatments. Nat Med. 2013;19(2):179-192. 10.1038/nm.3074 23389618

[93] Chen T, Wang Y, Hao Z, Hu Y, Li J. Parathyroid hormone and its related peptides in bone metabolism. Biochem Pharmacol. 2021;192:114669. 10.1016/j.bcp.2021.114669 34224692

[94] Wein MN, Liang Y, Goransson O, et al. SIKs control osteocyte responses to parathyroid hormone. Nat Commun. 2016;7(1):13176. 10.1038/ncomms13176 27759007 PMC5075806

[95] Guo J, Liu M, Yang D, et al. Suppression of Wnt signaling by Dkk1 attenuates PTH-mediated stromal cell response and new bone formation. Cell Metab. 2010;11(2):161-171. 10.1016/j.cmet.2009.12.007 20142103 PMC2819982

[96] Wan M, Yang C, Li J, et al. Parathyroid hormone signaling through low-density lipoprotein-related protein 6. Genes Dev. 2008;22(21):2968-2979. 10.1101/gad.1702708 18981475 PMC2577789

[97] Witcher PC, Miner SE, Horan DJ, et al. Sclerostin neutralization unleashes the osteoanabolic effects of Dkk1 inhibition. JCI Insight. 2018;3(11):e98673. 10.1172/jci.insight.98673 PMC6124404 29875318

[98] van Lierop AH, Moester MJ, Hamdy NA, Papapoulos SE. Serum Dickkopf 1 levels in sclerostin deficiency. J Clin Endocrinol Metab. 2014;99(2):E252-E256. 10.1210/jc.2013-3278 24302746

[99] Stolina M, Dwyer D, Niu QT, et al. Temporal changes in systemic and local expression of bone turnover markers during six months of sclerostin antibody administration to ovariectomized rats. Bone. 2014;67:305-313. 10.1016/j.bone.2014.07.031 25093263

[100] Florio M, Gunasekaran K, Stolina M, et al. A bispecific antibody targeting sclerostin and DKK-1 promotes bone mass accrual and fracture repair. Nat Commun. 2016;7(1):11505. 10.1038/ncomms11505 27230681 PMC4894982

[101] Anastasilakis AD, Polyzos SA, Avramidis A, Toulis KA, Papatheodorou A, Terpos E. The effect of teriparatide on serum Dickkopf-1 levels in postmenopausal women with established osteoporosis. Clin Endocrinol. 2010;72(6):752-757. 10.1111/j.1365-2265.2009.03728.x 19832854

[102] Gossiel F, Scott JR, Paggiosi MA, et al. Effect of teriparatide treatment on circulating periostin and its relationship to regulators of bone formation and BMD in postmenopausal women with osteoporosis. J Clin Endocrinol Metab. 2018;103(4):1302-1309. 10.1210/jc.2017-00283 29365099 PMC6457025

[103] Gatti D, Viapiana O, Idolazzi L, Fracassi E, Rossini M, Adami S. The waning of teriparatide effect on bone formation markers in postmenopausal osteoporosis is associated with increasing serum levels of DKK1. J Clin Endocrinol Metab. 2011;96(5):1555-1559. 10.1210/jc.2010-2552 21367927

[104] Idolazzi L, Rossini M, Viapiana O, et al. Teriparatide and denosumab combination therapy and skeletal metabolism. Osteoporos Int. 2016;27(11):3301-3307. 10.1007/s00198-016-3647-y 27250971

[105] Anastasilakis AD, Makras P, Pikilidou M, et al. Changes of circulating MicroRNAs in response to treatment with teriparatide or denosumab in postmenopausal osteoporosis. J Clin Endocrinol Metab. 2018;103(3):1206-1213. 10.1210/jc.2017-02406 29309589

[106] Yao GQ, Wu JJ, Troiano N, Insogna K. Targeted overexpression of Dkk1 in osteoblasts reduces bone mass but does not impair the anabolic response to intermittent PTH treatment in mice. J Bone Miner Metab. 2011;29(2):141-148. 10.1007/s00774-010-0202-3 20602130 PMC3457021

[107] Sridharan M, Cheung J, Moore AE, et al. Circulating fibroblast growth factor-23 increases following intermittent parathyroid hormone (1-34) in postmenopausal osteoporosis: association with biomarker of bone formation. Calcif Tissue Int. 2010;87(5):398-405. 10.1007/s00223-010-9414-8 20838781

[108] Drake MT, Srinivasan B, Mödder UI, et al. Effects of parathyroid hormone treatment on circulating sclerostin levels in postmenopausal women. J Clin Endocrinol Metab. 2010;95(11):5056-5062. 10.1210/jc.2010-0720 20631014 PMC2968729

[109] Ikeda T, Kaji H, Tamura Y, Akagi M. Once-weekly teriparatide reduces serum sclerostin levels in postmenopausal women with osteoprosis. J Orthop Sci. 2019;24(3):532-538. 10.1016/j.jos.2018.10.028 30573396

[110] Piemonte S, Romagnoli E, Bratengeier C, et al. Serum sclerostin levels decline in post-menopausal women with osteoporosis following treatment with intermittent parathyroid hormone. J Endocrinol Investig. 2012;35(9):866-868. 10.3275/8522 22842667

[111] Polyzos SA, Anastasilakis AD, Bratengeier C, Woloszczuk W, Papatheodorou A, Terpos E. Serum sclerostin levels positively correlate with lumbar spinal bone mineral density in postmenopausal women--the six-month effect of risedronate and teriparatide. Osteoporos Int. 2012;23(3):1171-1176. 10.1007/s00198-010-1525-6 21305266

[112] Tsai JN, Burnett-Bowie SM, Lee H, Leder BZ. Relationship between bone turnover and density with teriparatide, denosumab or both in women in the DATA study. Bone. 2017;95:20-25. 10.1016/j.bone.2016.11.009 27840301 PMC6941193

[113] Kobayakawa T, Kanayama Y, Hirano Y, Yukishima T, Nakamura Y. Therapy with transitions from one bone-forming agent to another: a retrospective cohort study on teriparatide and romosozumab. JBMR Plus. 2024;8(12):ziae131. 10.1093/jbmrpl/ziae131 PMC11601723 39605880

[114] Kramer I, Loots GG, Studer A, Keller H, Kneissel M. Parathyroid hormone (PTH)-induced bone gain is blunted in SOST overexpressing and deficient mice. J Bone Miner Res. 2010;25(2):178-189. 10.1359/jbmr.090730 19594304 PMC3153379

[115] Robling AG, Kedlaya R, Ellis SN, et al. Anabolic and catabolic regimens of human parathyroid hormone 1-34 elicit bone- and envelope-specific attenuation of skeletal effects in Sost-deficient mice. Endocrinology. 2011;152(8):2963-2975. 10.1210/en.2011-0049 21652726 PMC3138236

[116] Wu J, Cai XH, Qin XX, Liu YX. The effects of sclerostin antibody plus parathyroid hormone (1-34) on bone formation in ovariectomized rats. Z Gerontol Geriatr. 2018;51(5):550-556. Auswirkungen von Sclerostin-Antikorpern plus Parathormon (1-34) auf die Knochenbildung ovarektomierter Ratten. 10.1007/s00391-017-1219-1 28364259

[117] Lin J, Wu J, Sun S, et al. Combined antisclerostin antibody and parathyroid hormone (1-34) synergistically enhance the healing of bone defects in ovariectomized rats. Z Gerontol Geriatr. 2020;53(2):163-170. Kombinierter Antisklerostin-Antikorper und Parathormon (1-34) verbessern synergistisch die Heilung von Knochendefekten bei ovariektomierten Ratten. 10.1007/s00391-019-01685-2 31950363

[118] Sugiyama T, Torio T, Sato T, Matsumoto M, Kim YT, Oda H. Improvement of skeletal fragility by teriparatide in adult osteoporosis patients: a novel mechanostat-based hypothesis for bone quality. Front Endocrinol (Lausanne). 2015;6:6. 10.3389/fendo.2015.00006 25688232 PMC4311704

[119] Bodine PV, Seestaller-Wehr L, Kharode YP, Bex FJ, Komm BS. Bone anabolic effects of parathyroid hormone are blunted by deletion of the Wnt antagonist secreted frizzled-related protein-1. J Cell Physiol. 2007;210(2):352-357. 10.1002/jcp.20834 17044082

[120] Halloran BP, Bikle DD, Harris J, Tanner S, Curren T, Morey-Holton E. Regional responsiveness of the tibia to intermittent administration of parathyroid hormone as affected by skeletal unloading. J Bone Miner Res. 1997;12(7):1068-1074. 10.1359/jbmr.1997.12.7.1068 9200006

[121] Tanaka S, Sakai A, Tanaka M, et al. Skeletal unloading alleviates the anabolic action of intermittent PTH(1-34) in mouse tibia in association with inhibition of PTH-induced increase in c-fos mRNA in bone marrow cells. J Bone Miner Res. 2004;19(11):1813-1820. 10.1359/JBMR.040808 15476581

[122] Turner RT, Lotinun S, Hefferan TE, Morey-Holton E. Disuse in adult male rats attenuates the bone anabolic response to a therapeutic dose of parathyroid hormone. J Appl Physiol (1985). 2006;101(3):881-886. 10.1152/japplphysiol.01622.2005 16675609

[123] Edwards WB, Simonian N, Haider IT, et al. Effects of teriparatide and vibration on bone mass and bone strength in people with bone loss and spinal cord injury: a randomized, controlled trial. J Bone Miner Res. 2018;33(10):1729-1740. 10.1002/jbmr.3525 29905973

[124] Niimi R, Kono T, Nishihara A, Hasegawa M, Kono T, Sudo A. Efficacy of daily teriparatide treatment in low levels of walking state patients. Clin Drug Investig. 2017;37(6):551-557. 10.1007/s40261-017-0511-6 28247298

[125] Ma Y, Jee WS, Yuan Z, et al. Parathyroid hormone and mechanical usage have a synergistic effect in rat tibial diaphyseal cortical bone. J Bone Miner Res. 1999;14(3):439-448. 10.1359/jbmr.1999.14.3.439 10027909

[126] Roberts BC, Arredondo Carrera HM, Zanjani-Pour S, et al. PTH(1-34) treatment and/or mechanical loading have different osteogenic effects on the trabecular and cortical bone in the ovariectomized C57BL/6 mouse. Sci Rep. 2020;10(1):8889. 10.1038/s41598-020-65921-1 32483372 PMC7264307

[127] Sugiyama T, Saxon LK, Zaman G, et al. Mechanical loading enhances the anabolic effects of intermittent parathyroid hormone (1-34) on trabecular and cortical bone in mice. Bone. 2008;43(2):238-248. 10.1016/j.bone.2008.04.012 18539556

[128] Chow JW, Fox S, Jagger CJ, Chambers TJ. Role for parathyroid hormone in mechanical responsiveness of rat bone. Am J Phys. 1998;274(1):E146-E154. 10.1152/ajpendo.1998.274.1.E146 9458760

[129] Kim CH, Takai E, Zhou H, et al. Trabecular bone response to mechanical and parathyroid hormone stimulation: the role of mechanical microenvironment. J Bone Miner Res. 2003;18(12):2116-2125. 10.1359/jbmr.2003.18.12.2116 14672346

[130] Schulte FA, Marques FC, Griesbach JK, et al. Combined physical and pharmacological anabolic osteoporosis therapies increase bone response and mechanoregulation in female mice. Nat Commun. 2026;17(1):3759. 10.1038/s41467-026-70309-2 PMC13106859 41807441

[131] Capozza R, Ma YF, Ferretti JL, et al. Tomographic (pQCT) and biomechanical effects of hPTH(1-38) on chronically immobilized or overloaded rat femurs. Bone. 1995;17(4):S233-S239. 10.1016/8756-3282(95)00297-q 8579922

[132] Hagino H, Okano T, Akhter MP, Enokida M, Teshima R. Effect of parathyroid hormone on cortical bone response to in vivo external loading of the rat tibia. J Bone Miner Metab. 2001;19(4):244-250. 10.1007/s007740170027 11448017

[133] Rooney AM, Bostrom MPG, Dempster DW, Nieves JW, Zhou H, Cosman F. Loading modality and age influence teriparatide-induced bone formation in the human femoral neck. Bone. 2020;136:115373. 10.1016/j.bone.2020.115373 32330694 PMC7263665

[134] Meakin LB, Todd H, Delisser PJ, et al. Parathyroid hormone's enhancement of bones' osteogenic response to loading is affected by ageing in a dose- and time-dependent manner. Bone. 2017;98:59-67. 10.1016/j.bone.2017.02.009 28249797 PMC5404907

[135] Wehbi VL, Stevenson HP, Feinstein TN, Calero G, Romero G, Vilardaga JP. Noncanonical GPCR signaling arising from a PTH receptor-arrestin-Gbetagamma complex. Proc Natl Acad Sci USA. 2013;110(4):1530-1535. 10.1073/pnas.1205756110 23297229 PMC3557057

[136] Jongen JW, Willemstein-van Hove EC, van der Meer JM, et al. Down-regulation of the receptor for parathyroid hormone (PTH) and PTH-related peptide by PTH in primary fetal rat osteoblasts. J Bone Miner Res. 1996;11(9):1218-1225. 10.1002/jbmr.5650110905 8864895

[137] Mandl A, Burbelo PD, Di Pasquale G, et al. Parathyroid hormone resistance and autoantibodies to the PTH1 receptor. N Engl J Med. 2021;385(21):1974-1980. 10.1056/NEJMoa2109409 34788508 PMC9088239

[138] Fukayama S, Tashjian AH Jr, Bringhurst FR. Mechanisms of desensitization to parathyroid hormone in human osteoblast-like SaOS-2 cells. Endocrinology. 1992;131(4):1757-1769. 10.1210/endo.131.4.1396321 1396321

[139] Pun KK, Ho PW, Nissenson RA, Arnaud CD. Desensitization of parathyroid hormone receptors on cultured bone cells. J Bone Miner Res. 1990;5(12):1193-1200. 10.1002/jbmr.5650051202 1963732

[140] Wang B, Yang Y, Abou-Samra AB, Friedman PA. NHERF1 regulates parathyroid hormone receptor desensitization: interference with beta-arrestin binding. Mol Pharmacol. 2009;75(5):1189-1197. 10.1124/mol.108.054486 19188335 PMC2672812

[141] Bouxsein ML, Pierroz DD, Glatt V, et al. Beta-Arrestin2 regulates the differential response of cortical and trabecular bone to intermittent PTH in female mice. J Bone Miner Res. 2005;20(4):635-643. 10.1359/JBMR.041204 15765183 PMC1586119

[142] Sahbani K, Cardozo CP, Bauman WA, Tawfeek HA. Abaloparatide exhibits greater osteoanabolic response and higher cAMP stimulation and beta-arrestin recruitment than teriparatide. Physiol Rep. 2019;7(19):e14225. 10.14814/phy2.14225 31565870 PMC6766518

[143] Liu S, Jean-Alphonse FG, White AD, et al. Use of backbone modification to enlarge the spatiotemporal diversity of parathyroid hormone receptor-1 signaling via biased agonism. J Am Chem Soc. 2019;141(37):14486-14490. 10.1021/jacs.9b04179 31496241 PMC6930011

[144] Sato T, Verma S, Khatri A, et al. Comparable initial engagement of intracellular signaling pathways by parathyroid hormone receptor ligands teriparatide, abaloparatide, and long-acting PTH. JBMR Plus. 2021;5(5):e10441. 10.1002/jbm4.10441 33977197 PMC8101618

[145] Ebina K, Hirao M, Tsuboi H, et al. Effects of prior osteoporosis treatment on early treatment response of romosozumab in patients with postmenopausal osteoporosis. Bone. 2020;140:115574. 10.1016/j.bone.2020.115574 32777516

[146] Korff C, Adaway M, Atkinson EG, et al. Loss of Nmp4 enhances bone gain from sclerostin antibody administration. Bone. 2023;177:116891. 10.1016/j.bone.2023.116891 37660938 PMC10591883

[147] Holdsworth G, Greenslade K, Jose J, et al. Dampening of the bone formation response following repeat dosing with sclerostin antibody in mice is associated with up-regulation of Wnt antagonists. Bone. 2018;107:93-103. 10.1016/j.bone.2017.11.003 29129759

